# Preparation and Properties of GO/ZnO/nHAp Composite Microsphere Bone Regeneration Material

**DOI:** 10.3390/mi15010122

**Published:** 2024-01-11

**Authors:** Jiang Wu, Chunmei Wang, Shuangsheng Zhang, Ling Zhang, Jingshun Hao, Zijian Jia, Xiaomei Zheng, Yuguang Lv, Shuang Fu, Guoliang Zhang

**Affiliations:** 1 School of Stomatology, Jiamusi University, Jiamusi 154007, China; wujiangwj0126@163.com (J.W.); wcm13603693279@163.com (C.W.); zhangsszss2@163.com (S.Z.); 13603693279@163.com (L.Z.); 13359555272@163.com (J.H.); 17752712734@163.com (Z.J.); aaa643807758@163.com (X.Z.); 2 College of Pharmacy, Jiamusi University, Jiamusi 154007, China; lvyuguang@jmsu.edu.cn

**Keywords:** bone regeneration materials, graphene–zinc oxide–calcium phosphate composite microspheres, osteogenesis induction, antibacterial activity

## Abstract

The purpose of this study is to explore the possibility of using graphene–zinc oxide–hydroxyapatite (GO/ZnO/nHAp) composite microspheres as bone regeneration materials by making use of the complementary advantages of nanocomposites, so as to provide reference for the clinical application of preventing and solving bacterial infection after implantation of synthetic materials. Firstly, GO/ZnO composites and hydroxyapatite nanoparticles were synthesized using the hydrothermal method, and then GO/ZnO/nHAp composite microspheres were prepared via high-temperature sintering. The graphene–zinc oxide–calcium phosphate composite microspheres were characterized by X-ray diffraction (XRD), field emission scanning electron microscopy (FE-SEM), X-ray photoelectron spectroscopy (XPS), energy dispersion spectroscopy (EDS), water contact angle measurement, degradation and pH determination, and differential thermal analysis (DiamondTG/DTA). The biocompatibility, osteogenic activity, and antibacterial activity of GO/ZnO/nHAp composite microspheres were further studied. The results of the cell experiment and antibacterial experiment showed that 0.5% and 1% GO-ZnO-nHAp composite microspheres not only had good biocompatibility and osteogenic ability but also inhibited *Escherichia coli* and *Staphylococcus aureus* by more than 45% and 70%. Therefore, GO/ZnO/nHAp composite microspheres have good physical and chemical properties and show good osteogenic induction and antibacterial activity, and this material has the possibility of being used as a bone regeneration material.

## 1. Introduction

Bone repair is a therapeutic method to fill bone defects with bone regeneration materials to repair the morphology and restore part of the physiological function. The bone regeneration materials needed for treatment should have good biocompatibility, biological activity, bone induction, certain mechanical properties, and so on. At present, the commonly used bone regeneration materials mainly include autologous bone, allogenic bone, and xenogenic bone. Autogenous bone transplantation is the first choice for bone repair, but its application is affected by donor site infection, limited bone mass, and secondary surgical trauma; the source of allogeneic bone is limited, and it has the risk of immune rejection and ethics. In contrast, the synthetic bone substitute in xenogeneic bone has broader application prospects.

With the in-depth study of nano-biomaterials in the field of regenerative medicine, it has been found that the requirements of bone regeneration and repair are difficult to meet with a single material. Creating synergy through a composite of materials with different properties can give full play to the advantages and characteristics of all kinds of materials to a certain extent. In this study, we try to prepare a new bone regeneration material using graphene oxide–zinc oxide–hydroxyapatite (GO/ZnO/nHAp) composite microspheres, based on the complementary advantages of nanocomposites, in order to prevent and solve bacterial infection after the implantation of synthetic materials.

Hydroxyapatite (HAp) is a natural mineral form of calcium apatite, which is similar to the chemical composition of human bones and teeth, accounting for about 50% of bone weight. HAp has good biocompatibility and bioactivity, and has excellent bone conductivity and bone induction. It is usually used as a filler for bone replacement and bone tissue engineering or as a coating on implants to promote osseointegration [1]. HAp has the dual functions of focus filling and drug sustained release and plays an important role in the treatment of many bone diseases. However, the mechanical properties of HAp are not ideal, such as high brittleness, poor toughness, and easy fracture, making the material unsuitable for load bearing under load and torsion, limiting its application in the load-bearing part of the body [2]. In order to overcome the poor mechanical properties of HAp, carbon nanomaterials and metals have been added to hydroxyapatite to improve its mechanical properties [3].

Graphene is a new type of nanomaterial which is by far the thinnest nanomaterial found. The monolayer two-dimensional planar crystal formed by carbon atoms with sp^2^ hybrid orbitals has ideal physical and chemical properties and good biocompatibility and has been widely used in biomedicine and tissue engineering [4]. It has been confirmed that graphene (GN) and graphene oxide (GO) can promote the adhesion and proliferation of bone marrow mesenchymal stem cells and osteoblasts [5]. The excellent antibacterial activity of graphene oxide nanosheets was reported for the first time in 2010, which opened the era of GO and GO-based composites as antibacterial materials [6]. Some specific nanoparticles can be combined with graphene and the insertion of inorganic nanoparticles can reduce the interaction between graphene sheets and make composites have a synergistic effect. They have broad application prospects in the fields of catalysts, tissue engineering, biosensors, and so on.

Compared with ordinary zinc oxide, zinc oxide nanoparticles (ZnONPs) have a smaller particle size, larger surface area, and similar antibacterial properties to the traditional antibacterial agent silver [7]. Many studies have shown that a small number of ZnONPs can promote cell growth, proliferation, differentiation, tissue regeneration, angiogenesis, and bone integration [8].

**Figure 1:**
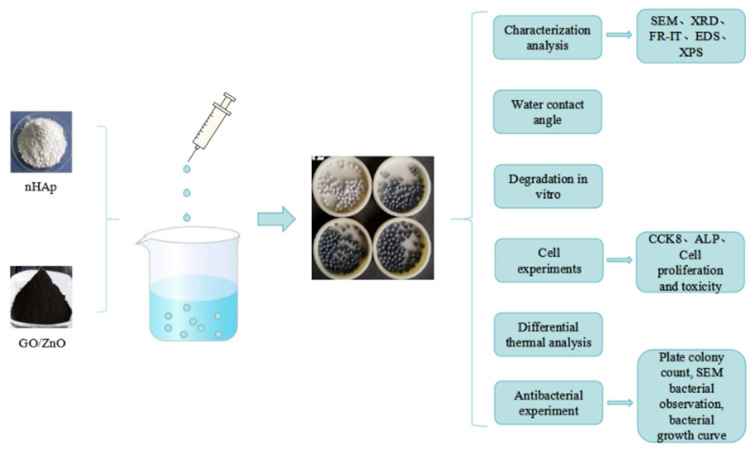
Flow chart of synthesis and performance research of GO/ZnO/nHAp composite microspheres.

Our research group has previously prepared graphene–zinc oxide (GO/ZnO) nanocomposites with antibacterial properties [9]. In this study, based on the excellent antibacterial properties of graphene nanocomposites, calcium phosphate microspheres supported by graphene nanocomposites were prepared by loading hydroxyapatite nanoparticles. It is predicted that the combination of calcium phosphate, graphene, and ZnONPs may produce both intrinsic antibacterial and bone-induced dual function. Figure 1 shows a flow chart of synthesis and performance research of GO/ZnO/nHAp composite microspheres.

## 2. Materials and Methods

### 2.1. Preparation of Hydroxyapatite Nanoparticles (nHAps)

Nanometer calcium phosphate particles were prepared using the molecular template hydrothermal method. According to the molar ratio of Ca and P in nHAp, which was 10:6, pure CaCl_2_ solid and Na_2_HPO_4_·12H_2_O solid were selected as the calcium source and phosphorus source, respectively, and the solution was prepared with EDTA-2Na as a template. We dissolved 0.012 mol Na2HPO_4_·12H_2_O and 0.02 mol EDTA-2Na in 30 mL deionized water and magnetically stirred it at 50 °C for 30 min and it fully dissolved. Then, the pH value of the above mixed solution was adjusted to 10 with NaOH solution (1 mol/L).

**Figure 2:**
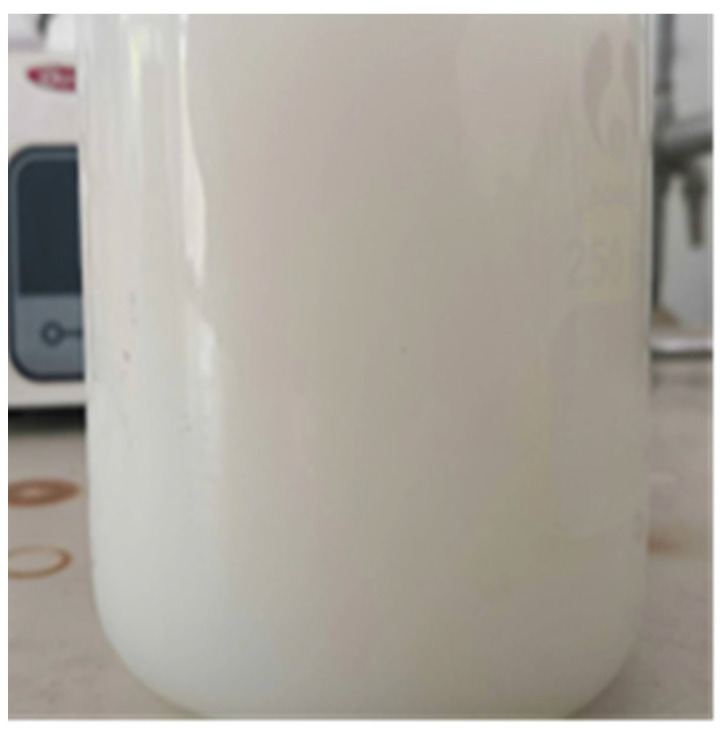
HAp synthesis process.

We dissolved 0.02 mol CaCl_2_ in 20 mL deionized water and stirred it until it fully dissolved. CaCl_2_ solution was added to the mixed solution of Na_2_HPO_4_·12H_2_O and EDTA-2Na at a constant drip rate and a milk-like suspension appeared (shown in Figure 2). During this process, NaOH solution was used to maintain a pH value = 10. After the solution was added, it was magnetically stirred at 60 °C for 2 h. The milky mixed solution was poured into the polytetrafluoroethylene inner tank, loaded into the hydrothermal synthesis reactor, and heated at 160 °C for 24 h. After cooling the reactor to room temperature, we removed the precipitation and put it into a centrifuge tube, rinsed and centrifuged the deionized water and anhydrous ethanol alternately (3 min, 3000 rmp) three times, freeze-dried it for 24 h, and then sealed and stored it away from light for later use.

### 2.2. Preparation of Graphene Oxide, Zinc Oxide, and Hydroxyapatite (GO-ZnO-nHAp) Composite Microspheres

Graphene oxide–zinc oxide (GO-ZnO) nanocomposites were provided by Professor Lv Yuguang’s team of the College of Pharmacy, Jiamusi University, Jiamusi, China. The GO-ZnO nanocomposites were synthesized via the hydrothermal method using aqueous zinc acetate solution and graphene oxide suspension as raw materials. In this process, graphene oxide (GO) was reduced to reduced graphene oxide (rGO). The composite microspheres were prepared using the ionic gel-drip method [10]. The preparation method was as follows: The prepared GO-ZnO nanocomposites and the prepared nHAp particles were dispersed in deionized water according to mass fractions of 0.5%, 1.0%, and 3.0% and ultrasonic mixing was performed to form a homogeneous suspension. Then, an equal amount of sodium alginate (SA) solution was added. After mixing well, the mixture was poured into a syringe. The mixed solution in the syringe was dropped into the CaCl_2_ aqueous solution. After the mixture has been completely added, the CaCl_2_ solution was cross-linked, and then the CaCl_2_ solution was poured out and cleaned with deionized water. After drying, the corresponding microspheres were sintered in a multifunctional vacuum sintering furnace.

**Figure 3:**
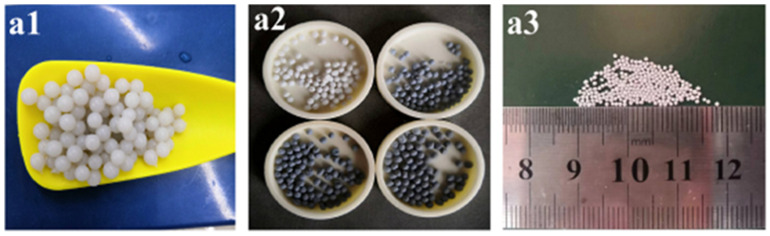
Macroscopic appearance of composite microspheres. (**a1**) Microspheres synthesized by ionic gel-drip method. (**a2**) Four groups of dried microspheres. (**a3**) After drying, the diameter of microspheres is about 1 mm.

Taking pure nHAp microspheres in the control group as an example, a certain amount (0.2 g) of nHAp was dissolved in 15 mL deionized water; this was subjected to ultrasonic shock for 5 min and magnetic stirring at 50 °C for 10 min, and after the solution had been evenly stirred, an equal amount of SA solution was added which was continuously stirred for 30 min. After mixing well, the HAp-SA mixture was poured into a 20 mL syringe. A total of 11.10 g calcium chloride (CaCl_2_) was dissolved in 1000 mL deionized water to obtain 0.1 mol/L of CaCl_2_ aqueous solution, and the HAp-SA mixed solution loaded in the syringe was dropped into the CaCl_2_ aqueous solution. After the HAp-SA mixture had been completely added, it continued to be subjected to cross-link calcification in CaCl_2_ aqueous solution for 45 min; then, the CaCl_2_ solution was poured out to obtain unsintered composite microspheres (see Figure 3(a1)) and cleaned with deionized water three times. Finally, it was dried overnight in a vacuum oven at 30 °C (see Figure 3(a3)) and then sintered at 1000 °C at a heating rate of 5 °C/min, rising from room temperature to 400 °C, held for 1 h, and sintered at the highest temperature for 15 min to obtain hydroxyapatite microspheres (see Figure 3(a2)).

### 2.3. Analysis and Characterization

#### 2.3.1. nHAp Characterization

##### Field Emission Scanning Electron Microscope (FE-SEM)

An FE-SEM was mainly used to analyze the microstructure of the sample. Because the nHAp of the sample was not conductive, it was necessary to spray gold on the surface of the sample before testing.

##### X-ray Diffraction (XRD)

The crystallinity of the sample was analyzed by XRD. We filled the groove of the sample holder with nHAp, gently scraped and flattened it with a slide, trimmed off the excess sample, and collected it. Test conditions: Cu-Kα radiation (voltage 36 KV, current 30 mA), in the 2θ zone 5~80° range, scanning speed of 4°/min maintained, step size of 0.02°.

##### Fourier Transform Infrared (FT-IR)

The absorption spectrum of the measured sample was compared with the standard spectrum using FT-IR, which can identify a compound and determine its molecular structure; then, we performed functional group analysis of the sample, verified the chemical composition of the sample, and determined the substance. A potassium bromide tablet was used for sample preparation and the scanning spectrum recording range was 4000–400 cm^−1^. The prepared nHAp particle sample and dried potassium bromide were ground into powder using an agate mortar, and the mixed powder was pressed with a tablet press for the FT-IR test.

#### 2.3.2. Characterization of GO-ZnO-nHAp Composite Microspheres

##### X-ray Diffraction (XRD)

The prepared composite microsphere sample was ground into a powder using an agate mortar, the groove of the sample holder was filled, and the excess sample was gently scraped and flattened with a slide. The excess sample was hung and collected. Test conditions: Cu-Kα radiation (voltage 36 KV, current 30 mA), in the 2θ zone 5~80° range, scanning speed of 4°/min maintained, step size of 0.02°.

##### Field Emission Scanning Electron Microscope (FE-SEM)

The morphology of the microsphere samples was studied using FE-SEM. Because the sample was not conductive, it was necessary to spray gold on the surface of the sample before testing.

##### X-ray Photoelectron Spectroscopy (XPS) 

XPS can quantitatively and qualitatively analyze the elements of a complex and can analyze a solid surface, including the elemental composition, valence state, and surface energy distribution. In this experiment, an X-ray photoelectron spectroscopy analyzer (PHI5000 Versaprobe III, Ulvac-Phi, Chigasaki, Japan) was used to analyze the valence state and content of elements of the composite microspheres. The voltage was 16 KV, the current was 14.9 mA, and the charging calibration was carried out with C1s = 284.8 eV as the standard.

##### Energy Dispersive Spectrometer (EDS)

A high-performance EDS attached to an FE-SEM can analyze the composition of a certain microregion of a sample to conduct a quantitative analysis.

##### Water Contact Angle Measurement

The composite microsphere sample was dried in a vacuum at 60 °C for 24 h, pressed using a tablet press, and then glued onto the slide. The water contact angle of the composite microsphere was measured using a contact angle tester. Samples in each group were tested 3 times, and the average value was the test result.

##### Determination of Degradation Performance and pH Value

In vitro degradation performance was measured by placing the material in Phosphate-Buffered Saline (PBS). A 40 mL plastic centrifuge tube was used, the microspheres of different proportions were weighed (W_0_) and placed in it, and then the PBS (30 mL) solution was added to the water bath at 37 °C. During the immersion, the pH value of the solution was recorded using a pH meter. Samples were taken out at the set time point on day 1, 3, 5, 7, 14, 21, and 28, washed with distilled water 3 times, dried at 37 °C until their mass remained stable, weighed (W_t_), and recorded. The weight loss rate of microspheres before and after immersion was calculated as follows:

Microsphere weight loss rate = (W_0_ − W_t_)/W_0_ × 100%.

##### Differential Thermal Analysis (TG-DSC)

The thermal stability of the composite was analyzed using differential thermal analysis. The mass, temperature difference, and heat change in the sample were recorded during the heating process. Using a nitrogen atmosphere, the heating range was 30~1100 °C and the heating rate was 10 °C/min.

### 2.4. Cell Experiments

#### 2.4.1. Cell Culture

**Figure 4:**
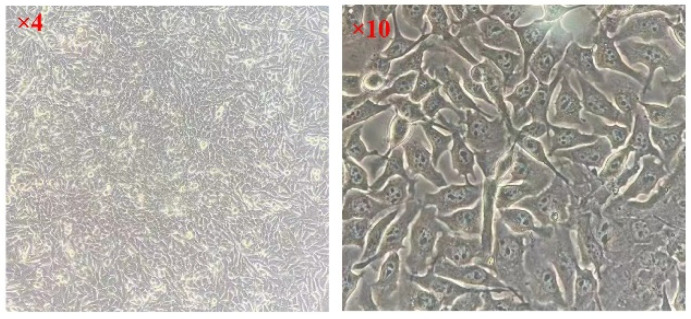
Growth state of MC3T3-E1 cells.

The frozen MC3T3-E1 cells (provided by the School of Basic Medicine of Jiamusi University, Jiamusi, China) were taken out of the tube and placed in a water bath at 37 °C for oscillating melting. They were then transferred into sterile culture bottles. The cells were cultured in a constant temperature incubator at 37 °C, with 5% CO_2_ and 95% humidity, and replaced with fresh DMEM 48 h later; the growth state of the cells was observed. The growth state of cells after resuscitation is shown in Figure 4.

#### 2.4.2. Preparation of the Extract

According to the ISO10993-12:2021 [11] recommended method, pure nHAp microspheres were used as an example to prepare the extract. DMEM containing 10% FBS, which was the extract of the microsphere sample, was added in proportion (sample mass (g)/medium size (mL) = 0.2 g/mL). It was placed in a sterile centrifuge tube and stored in a refrigerator at 4 °C for 7 days.

#### 2.4.3. Preparation of Control Group Solution

Cells cultured with culture medium were the negative control group; only the culture medium without cells was added as a blank control group.

#### 2.4.4. DMEM Osteogenic Induction Medium

DMEM osteogenic induction medium is composed of the following: dexamethasone 10 µg, vitamin C 25 mg, β-sodium glycerophosphate 1.08 g, and DMEM 500 mL.

#### 2.4.5. Cytotoxicity Was Detected by CCK-8 Method

After the cells were attached to the wall, the original medium in the blank control group, the negative control group, and the experimental group were discarded and washed with 200 μL PBS. After the PBS was sucked out, the sample extract was added to each well of the experimental group. DMEM containing 10% FBS was added to the blank control group and the negative control group. The 96-well cell culture plates were then cultured in a constant temperature cell incubator at 37 °C and 5% CO_2_. The solution was changed every 2 d for 1, 3, 5, and 7 days, and 10 μL CCK-8 reagent was added to each well at each time point. OD values of each well on a 450 nm wavelength were detected using an enzyme-labeler. The cell survival rate (%) was calculated according to the absorbance values of each group and the formula was as follows:

Cell survival rate (%) = (experimental group − blank control group)/(negative control group − blank control group) × 100%.

#### 2.4.6. Alkaline Phosphatase (ALP) Detection in Cells Cultured with the Material Extract

MC3T3-E1 was inoculated into a 6-well plate, and after 24 h of cell adhesion, the old medium was removed and replaced with the osteogenic induction solution of the extract of the material samples of each group, which was cultured in an incubator. The solution was changed the next day and the ALP activity was detected at the time points of 7 and 14 days.

ALP activity in cultured cells (Kim’s unit/gprot) = (measured OD value-blank OD value)/(standard OD value-blank OD value) × phenol standard concentration (0.02 mg/mL)/sample protein concentration (gprot/mL).

### 2.5. Antibacterial Performance Test

#### 2.5.1. *E. coli* Used as an Example

After routine recovery, *E. coli* strain was inoculated on solid medium and cultured at 37 °C for 24 h. A single colony with good growth status and obvious characteristics was selected and inoculated into BHI (broth) medium. The bacterial solution grew to the logarithmic growth stage and was centrifuged at room temperature (220 rpm/min for 4 min) and the supernatant was discarded. The bacterial precipitates were washed twice with sterile saline and then re-suspended in sterile saline. The concentration of bacterial solution was adjusted to OD600 ≈ 0.7 using an ultraviolet spectrophotometer (UV-1601, Beijing Beifen Ruili, Beijing, China).

#### 2.5.2. Plate Colony Counting Method

The antibacterial activity of the samples against Gram-negative *E. coli* (ATCC25922) and Gram-positive *Staphylococcus aureus* (*S. aureus*, ATCC6538) was studied using the plate colony counting method. We took *E. coli* as an example to describe the process.

The microsphere samples were sterilized via ultraviolet irradiation. A total of 0.2 g composite microspheres was mixed with 1 mL of material PBS, 0.2 mL of bacterial suspension (OD600 ≈ 0.7), and 5 mL of nutrient broth medium (NB) and cultured at 37 °C for 24 h to form a mixed solution. We diluted the mixture after co-culture: 4.5 mLPBS was injected into a glass test tube and then 0.5 mL of the mixture was added. After the mixture had been evenly mixed, the mixture was diluted 10 times successively and 50 μL of the final diluted solution was evenly coated on the surface of the BHI solid medium plate and cultured at 37 °C for 24 h (S.Ureus culture for 18 h) to observe colony formation; colonies were counted and photos were taken. The result was multiplied by the corresponding dilution, which was the number of viable bacteria under this condition, in CFU/mL. The experiment was repeated three times. We used the following formula to calculate the bactericidal rate R:

R = [(C_0_ − C_t_)/C_0_] × 100%

(C_0_: The average colony number of living bacteria in the control group, C_t_: The average colony number of living bacteria in the experimental group).

#### 2.5.3. Observation of Bacterial Morphology

We obtained five Petri dishes. A total of 3 mL of 1 × 10^7^ CFU/mL bacterial solution and 3 mL culture solution were placed in the Petri dishes, along with 0.12 g composite microspheres of different proportions and the prepared 10 × 10 mm glass sheets. The group without composite microspheres was the control group and the group with composite microspheres was the experimental group (4 groups). The composite microspheres were placed in bacterial solution and incubated at 37 °C for 48 h under aerobic culture; then, the glass pieces were removed, washed with PBS solution 3 times, fixed with 2.5% glutaraldehyde, dehydrated with ethanol gradient (30%, 50%, 70%, 80%, 90%, 95%, 100%), dried, sprayed with gold, and observed under a scanning electron microscope.

#### 2.5.4. Effects of Composite Microspheres on the Growth State of Bacteria

*E. coli* suspensions were prepared via overnight culture and the concentration of the suspensions was adjusted to about 1 × 10^6^ CFU/mL. Antibacterial solution containing composite microspheres of different proportions was prepared using BHI liquid medium and normal BHI liquid medium without composite microspheres was used in the control group. We mixed the bacterial solution with the above antibacterial solution evenly and added 200 μL per well into the 96-well plate. The above mixture was cultured in a constant-temperature oscillating incubator at 37 °C for 24 h, during which the absorbance (OD) value of each hole at 600 nm was measured every 2 h, and then the bacterial growth curve was plotted using time as the horizontal coordinate and the OD (600) value as the vertical coordinate. There were three parallel experiments for each concentration.

### 2.6. Statistical Analysis

The measurement data were expressed as mean ± SD. SPSS22.0 software was used and a *t*-test was used for data comparison between the two groups; a one-way ANOVA test was used for data comparison between multiple groups. *p* < 0.05 was considered to be statistically significant.

## 3. Results and Discussion

### 3.1. Characterization of Nano-Hydroxyapatite Particles (nHAps)

**Figure 5:**
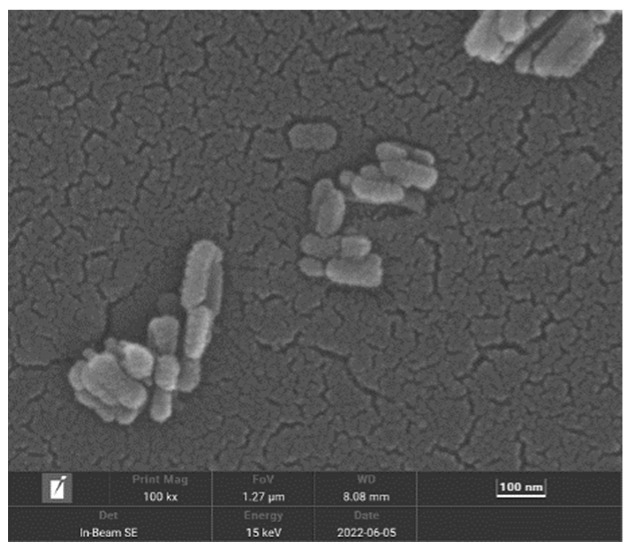
Scanning electron microscopy of nHAp.

**Figure 6:**
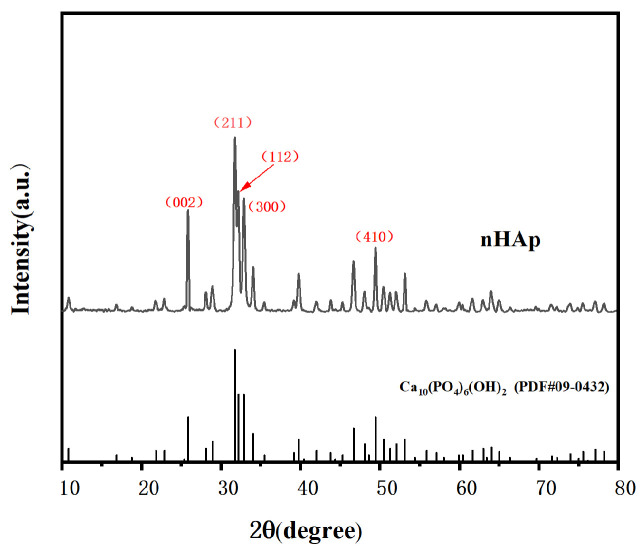
XRD pattern of nHAp.

**Figure 7:**
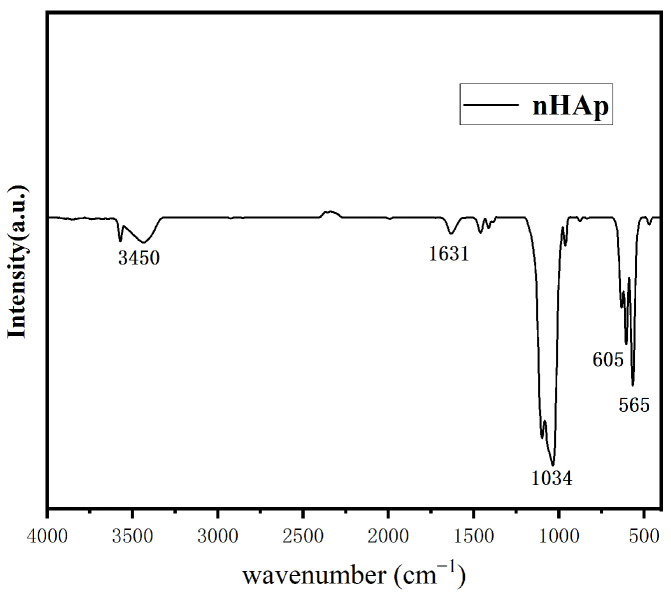
FTIR pattern of nHAp.

Figure 5 shows the morphology of the final product prepared at pH = 10. As shown in Figure 5, in an alkaline environment, the shape of the final product is short and rod-like, with a diameter of about 50 nm and a length of about 100–150 nm, which is on the nanoscale. It is considered that this nanoscale material is suitable for transport in vivo and can be taken up by cells [12]. Figure 6 shows the XRD pattern of the prepared nHAp nanorods. In the figure, nHAp crystal faces such as (002), (211), (112), (300), (202), and (410) have characteristic diffraction peaks. Among them, the four crystal planes (002), (211), (112) and (300) have the strongest diffraction and are the main diffraction peaks, and no other impurity-phase diffraction peaks appear, which proves that HAp has high crystallinity and a relatively complete crystal form [13]. The results show that the synthesized products have good crystallinity, which is consistent with the diffraction peaks of standard cards (Ca_10_(PO_4)6_(OH_2_), JCPDS No. 09-0432). Figure 7 is the FTIR spectrum of nHAp. It can be seen that 1034 cm^−1^ and 565 cm^−1^ are PO_4_^3-^ stretching vibration peaks, while 3450 cm^−1^ and 1631 cm^−1^, respectively, correspond to -OH and H_2_O molecular stretching vibration peaks, which are consistent with the PO_4_^3−^, -OH, and H_2_O molecular stretching vibration peaks of nHAp. The results show that nHAp was successfully synthesized. This is consistent with the XRD results. Because HAp has the capability of bone binding, artificial bones composed of HAp are widely used as bone grafts. The preparation processes of HAp include the chemical precipitation method, sol–gel method, hydrothermal method, microwave radiation, solid state reaction, etc., as well as the utilization of animal, plant, and marine resources to prepare HAp nanoparticles with different morphological features and chemical compositions [14]. Among them, the hydrothermal method has the advantages of environmental protection, high crystallinity of synthesized hydroxyapatite, and Hap morphology, which can be controlled by adjusting the pH value, time, and temperature of a reaction system [12]. Since the biological reaction of HAp nanoparticles is largely dependent on their size and morphology, the preparation of nanoparticles with nanostructures is of great significance to meet growing demand in the biomedical field.

### 3.2. Characterization of GO-ZnO-nHAp Composite Microspheres

In this study, nHAp were mixed with the prepared GO-ZnO nanocomposites with mass fractions of 0.5%, 1.0%, and 3.0%, sodium alginate was used as the adhesive, and they were combined using atmosphere sintering technology. At the micro level, the essence of sintering and bonding of materials is the same, whereby both of which are a process of replacing the “solid-solid” interface and forming metallurgical bonding by eliminating the “solid-gas” interface between materials [15]. This is because high-temperature sintering can not only make the powder sample achieve fusion between particles in a very short time, but can also compress the particles, thus densifying the material [16]. Relevant research results show that bone repair materials contain pores with an appropriate volume fraction, which can provide channels and growth space for the growth of cells, fiber tissue, and bone tissue, increase the contact surface area between tissue fluid and composite scaffolds, and determine the degree and speed of bone growth by affecting metabolism, nutrient transport, and blood vessel growth, thus speeding up the process of bone repair [17]. Therefore, in this study, sodium alginate was used as a bonding agent and pore-causing agent to temporarily link GO-ZnO nanocomposites with nHAp, and then sodium alginate was pyrolyzed by atmospheric sintering, leaving pores inside the microspheres.

**Figure 8:**
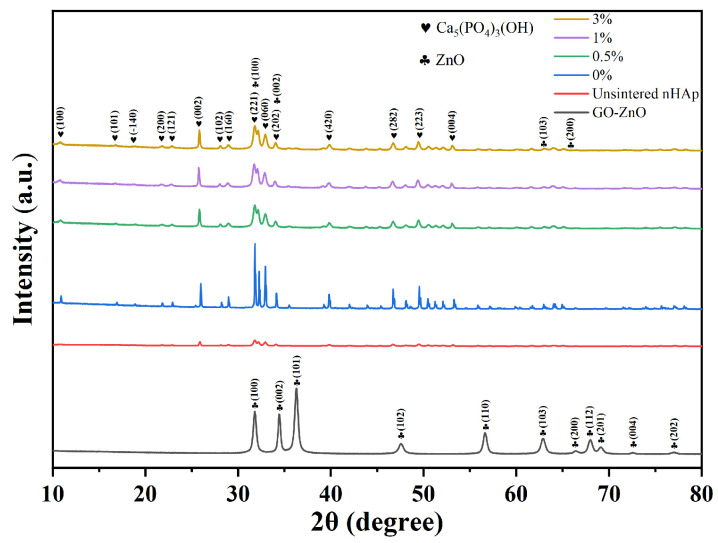
XRD pattern of composite microspheres.

Figure 8 shows the XRD pattern of composite microspheres after sintering at a high temperature. There are no peaks of GO, ZnO, or GO-ZnO in the XRD patterns of the composite microspheres with Go-zno spectra, which may be due to the low content of Go-zno compared with the bulk. The XRD pattern of composite microspheres mainly shows nHA peaks. The XRD pattern of the 0% group (pure nHAp) shows that the diffraction peaks of each phase sharpened and narrowed after sintering, indicating that the crystallinity increased. After sintering, the intensity of the characteristic diffraction peak (300) is stronger, while that of the characteristic diffraction peak (002) is weaker. Compared with the 0% group, the diffraction peaks of the 0.5%-, 1%-, and 3%-sintered groups are wider, which indicates that the addition of GO-ZnO changes the structure of the nHAp phase and makes the crystallinity of the grains lower. It is worth noting that the GO-ZnO-nHAp composite microsphere composite has no stray peaks in the XRD pattern, indicating that a relatively pure composite microsphere was synthesized.

**Figure 9:**
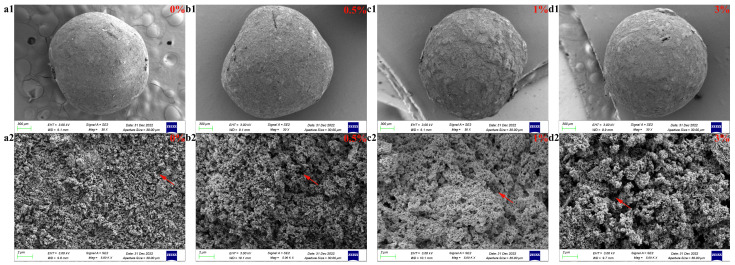
SEM images of composite microspheres: surface and sectional view. Surface diagram of each group of microspheres (**a1**,**b1**,**c1**,**d1**). Cross-sectional view of each group of microspheres (**a2**,**b2**,**c2**,**d2**).

Before sintering at a high temperature, the surface of GO-ZnO-nHAp composite microspheres is smooth, the shape is spherical, the particle size is relatively uniform (Figure 3(a1)), and the diameter is about 1 mm (Figure 3(a3)). Due to the different proportions of GO-ZnO nanomaterials, the color of the microspheres changes from white to dark gray (Figure 3(a2)). After sintering at a high temperature, the volume shrinks, and SEM observation shows that the microspheres are spherical or quasi-spherical. With an increase in the GO-ZnO ratio, the surface holes gradually increase from smooth to rough. The cross-section of the microspheres shows that there are a lot of pores in the microspheres, and with the increase in the GO-ZnO ratio, the pores in the microspheres gradually increase. The water and sodium alginate contained in the microspheres volatilize and pyrolyze at a high temperature, which leads to a change in the volume and morphology of the microspheres. The red arrow in Figure 9 indicates the pores left inside the microspheres due to the pyrolysis of sodium alginate.

**Figure 10:**
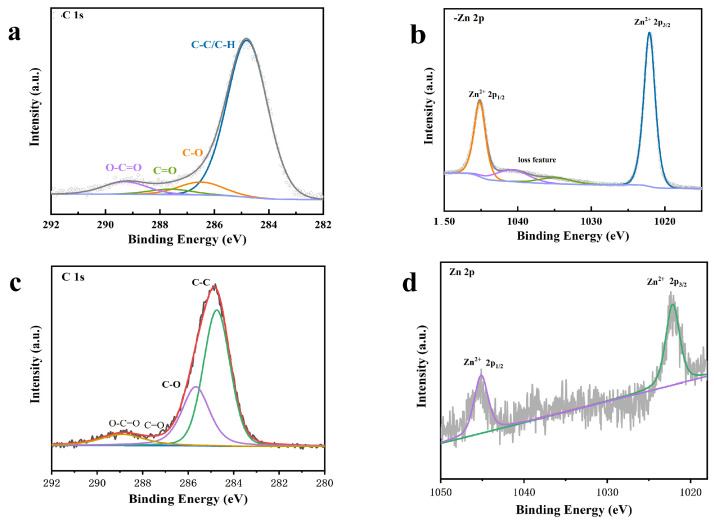
XPS spectra. (**a**,**b**) are the XPS spectra of the 1% group composite microspheres: (**a**) C 1 s; (**b**) Zn 2 p. (**c**,**d**) are the prepared GO-ZnO XPS spectra: (**c**) C 1 s; (**d**) Zn 2 p.

The composition and electronic structure of materials, surface chemical bonds, and chemical interactions can generally be detected by XPS studies. The characteristic energy peaks of zinc and carbon in the composite are C1s and Zn2p, respectively. Figure 10 shows the XPS spectra of GO-ZnO and GO-ZnO-nHAp 1% composite microspheres. Figure 10a shows the C1s sub-peak fitting diagram of XPS for rGO. It can be seen from the diagram that there are three obvious Gaussian fitting peaks at 284.4 eV, 286.0 eV, and 287.7 eV, which are attributed to C-C, C-O(epoxy), and C=O(aromatic), respectively. Among them, the C-C functional group is the chemical bond formed by the carbon–carbon atoms of the GO carbon skeleton SP2 hybridization. It can be seen from Figure 10a that the oxygen-containing functional groups C-OH and C=O are significantly reduced after the hydrothermal reaction compared with graphene oxide, indicating that graphene oxide in the composite is reduced graphene oxide (rGO), which is consistent with the state of graphene in the GO-ZnO composite before sintering (Figure 10c). It also shows that graphene oxide did not undergo chemical changes after high-temperature sintering. In the Zn2p spectrum, there are two strong peaks in Figure 9(d1,d2) at 1022.5 eV and 1045.5 eV, corresponding to the binding energies of Zn 2p3/2 and Zn 2p1/2, respectively. These peaks are consistent with those of pure ZnO, indicating that zinc in the composite exists in an oxidation state in the form of Zn^2+^ [18].

**Figure 11:**
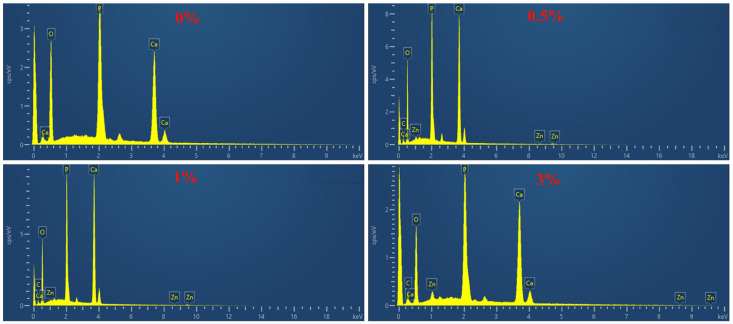
EDS diagram of composite microspheres.

In order to determine the chemical composition of the composite microspheres, EDS detection was performed on the microspheres, as shown in Figure 11. According to the EDS spectra, the main elements of the composite microspheres are C, O, Zn, Ca, and P.

**Figure 12:**
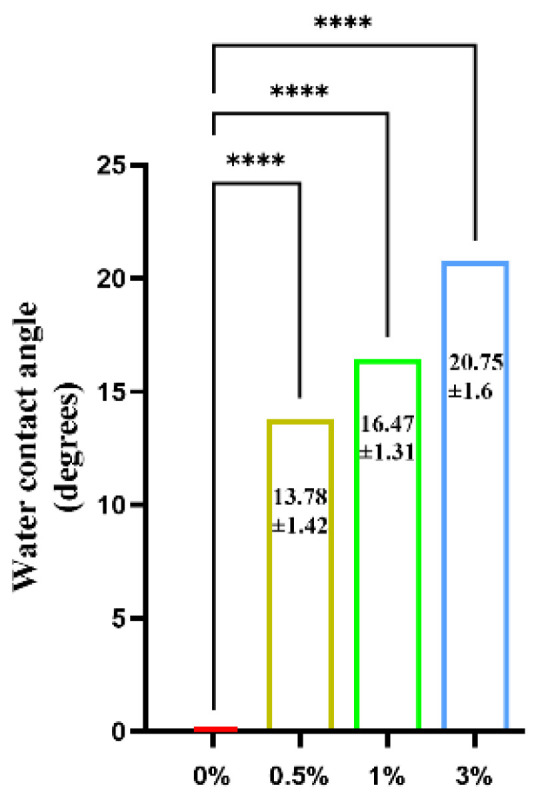
Water contact angle test of composite microspheres.****, *p* < 0.0001, *n* = 3.

The water contact angle test results of the GO-ZnO-nHAp composite microspheres show that the contact angle of 0% (pure nHAp) is 0°. With the addition of GO-ZnO composite materials, the water contact angle of GO-ZnO-nHAp composite microspheres gradually increases (see Figure 12 for details), and the contact angles of 0.5%, 1%, and 3% are 13.78 ± 1.42, 16.47 ± 1.31, and 20.75 ± 1.6. Compared with 0% (pure nHAp), the contact angles of 0.5%, 1%, and 3% have obvious statistical significance, indicating that the composite has good hydrophilicity.

**Figure 13:**
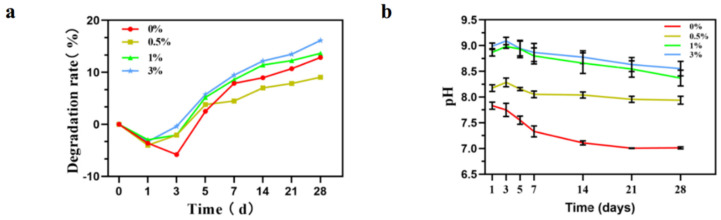
Changes in composite microspheres in PBS solution. (**a**) Degradation rate curve; (**b**) pH value change.

Figure 13a shows the weight changes in composite microspheres soaked in PBS solution for different times. With an increase in soaking time, the mass of composite microspheres decreases and the surface becomes rough, indicating that the microspheres gradually degraded. As can be seen from the figure, the degradation trend of the four groups of samples remained the same, and the first three days were all weight gain processes. Weight gain was caused by the surface activity of the microsphere which deposited the active ions in PBS solution such as Ca^2+^ and HPO_4_^2−^ on its surface, thus making it gain weight. Subsequently, the microsphere began to degrade slowly because the microsphere itself contained hydroxyapatite. Microspheres continuously release Ca^2+^ and the PBS solution continuously updates to provide Ca^2+^ to the deposition, so that the degradation rate and the deposition rate maintain a certain equilibrium state. It can be seen from the degradation trend of the graph that the degradation of microspheres is relatively slow.

The change in pH value during soaking is shown in the figure (Figure 13b). The pH evolution trend of the composites was similar during immersion in PBS solution. A rapid increase in pH was observed in the initial 3 days, and after which a relatively stable state was reached. After soaking for 28 days, the pH value of the 0% group reached about 7.0. The pH value of the 0.5% group reached about 8.0; the pH value of the 1% group reached about 8.5; and the pH of the 3% group reached about 8.6.

**Figure 14:**
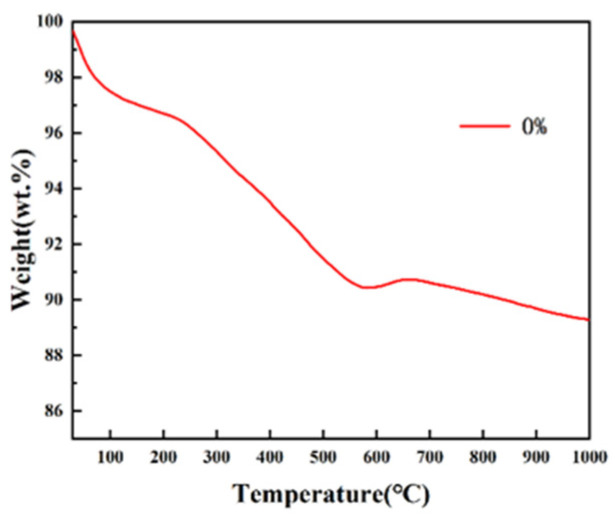
TG curves of pure nHAp microspheres.

In order to determine the sintering temperature and time of the composite microspheres at a high temperature to ensure the complete pyrolysis of sodium alginate, thermogravimetric (TG) analysis was performed on 0% hydroxyapatite microspheres. Thermogravimetry measures the weight of a substance as it changes with temperature during a set temperature rise. As shown in Figure 14, it can be seen from the TG curve that the mass loss of pure nHAp microspheres added with sodium alginate can be divided into three main processes. At the stage of 30~150 °C, the evaporation of water in the physical adsorption state of the microsphere causes the quality to decline and the mass of the product reduces by about 3%. At the stage of 150~500 °C, the mass loss is about 7% due to the pyrolysis of sodium alginate. After 500 °C, the TG curve becomes gentle, indicating that the pyrolysis of sodium alginate is complete. The TG curve at 600~1000 °C is relatively gentle and the mass of microspheres is relatively constant and slightly changes, which is considered to be caused by the loss of strongly bound water in nHAp [19]. In the test temperature range, the percentage of weight loss of the microspheres decreased by 12.05%, which means that the composite microspheres have excellent thermal stability.

### 3.3. Cytological Test Results

**Figure 15:**
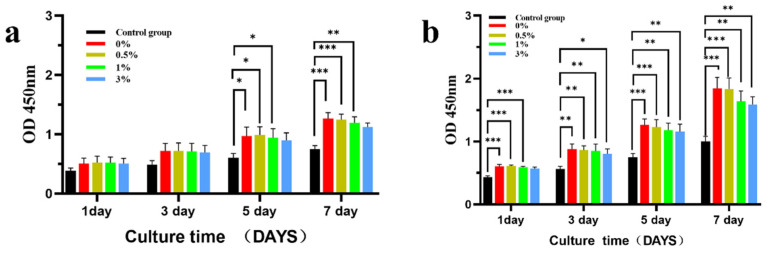
The effect of different concentrations of composite microsphere extracts on the proliferation of MC3T3-E1 cells was detected by CCK-8, where (**a**) was 100% extract and (**b**) was 50% extract. * *p* < 0.05; ** *p* < 0.01; *** *p* < 0.001; *n* = 3.

Figure 15 shows the effects of the extracts of different groups of composite microspheres on the proliferation of MC3T3-E1 cells. MC3T3-E1 cells were co-cultured with different concentrations of GO-ZnO-nHAp composite microspheres and the growth state of the cells was good. After 100% extract was co-cultured with MC3T3-E1 cells, there was no statistical difference in cell proliferation between the 1st and 3rd day and the negative control group at the same time, but there was a statistical difference in cell proliferation between the 0%, 0.5%, and 1% groups on the 5th and 7th days, and the statistical difference was significant on the 7th day. The results showed that 0%, 0.5%, and 1% composite microspheres had a good proliferation effect on cells (Figure 15a). Compared with the negative control at the same time, the 50% concentration composite microsphere extract has obvious statistical differences at each time point (Figure 15b). This shows that each group of GO-ZnO-nHAp composite microspheres has no obvious inhibitory effect on cell proliferation and has good cell biocompatibility.

**Figure 16:**
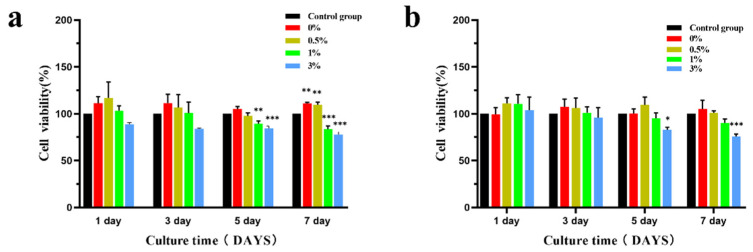
Effects of different concentrations of composite microsphere extracts on the activity of MC3T3-E1 cells ((**a**) is 100% extract, (**b**) is 50% extract); * *p* < 0.05; ** *p* < 0.01; *** *p* < 0.001; *n* = 3.

Figure 16 shows the relative proliferation rate of MC3T3-E1 cells after co-culture of microspheres with 100% and 50% concentration extracts. Compared with the negative control group at the same time, MC3T3-E1 cells in the extracts of the four experimental groups all grew well and did not show obvious cytotoxicity. There was no statistical difference in cell activity between the 100% and 50% concentration extracts and the negative control group at the same time on the 1st and 3rd day. The cell activity in the 100% concentration extracts was statistically significant in the 1st and 3rd groups on the 5th day, and the cell activity in the four groups of microsphere extracts on the 7th day was statistically significant. The cell activity in the 50% concentration extract was statistically different on the 5th and 7th day only in the 3% group. According to the ISO10993-5 standard [20] (Table 1), the cytotoxicity of the composite extracts of the four experimental groups was 0–1, showing good biocompatibility.

**Figure 17:**
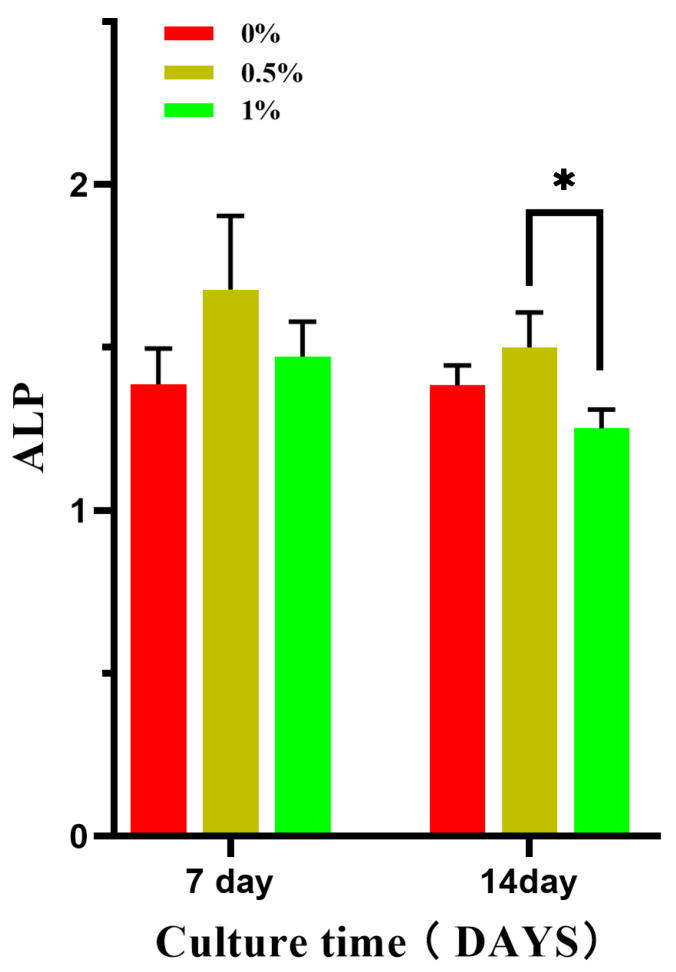
Effect of composite microsphere extract on the expression of ALP in MC3T3-E1 cells; * *p* < 0.05; *n* = 3.

According to the CCK-8 detection results, 0%, 0.5%, and 1% composite microspheres were selected for alkaline phosphatase level detection. Figure 17 shows the expression of alkaline phosphatase at day 7 and day 14 after 100% extracts of different composite microspheres were co-cultured with MC3T3-E1 cells. ALP is a marker of early osteogenic differentiation and plays an important role in the differentiation and maturation of osteoblasts, thus promoting bone formation [21,22]. The effect of alkaline phosphatase on the differentiation of MC3T3-E1 cells was measured at different time points. The level of alkaline phosphatase in the 0.5% group was higher than that in the 0% and 3% groups on the 7th and 14th day and there was a statistical difference between the 0.5% and 1% groups on the 14th day, but there was no significant difference between the other groups. 

In terms of bone regeneration, hydroxyapatite (HAp), tricalcium phosphate (TCP), bioactive glass (BG), and other bioceramics can promote the differentiation and proliferation of osteoblasts and have been widely used in dental and orthopedic fields. The inorganic component of bone ECM (extracellular matrix) is composed of nanoscale calcium phosphate (CaP) crystals similar to hydroxyapatite. Given the natural composition of bone, CaP material is a reasonable choice as a biomaterial. In fact, CaP ceramics exhibit good biological properties because they are able to form chemical binding boundaries with bone [23]. Because their chemical properties are similar to those of natural bone minerals, these bioceramics are highly biocompatible. Compared with micron-scale bulk HAp, HAp with a nanostructure showed better biological properties, including high drug loading ability, excellent osteogenesis and bone integration ability, and unique selective anticancer ability. The osteogenic process includes the interaction of osteoblasts and osteoclasts with bioceramics to repair or heal fractures [24]. The composite microspheres contain hydroxyapatite, and Ca^2+^ ions can be released during the preparation of the extract. The increase in Ca^2+^ ion concentration will affect the function of osteoblasts and promote the osteogenic differentiation of MC3T3-E1. However, some studies have shown that HAp has different biological activity on different cells. MaX et al. tested cell viability with pure HAp in cancer cells (HeLa) and normal cells (L02), respectively. The results showed that CaP granules were not toxic to normal cells, but had specific toxicity to tumor cells. Lower pH in tumor cells may accelerate the Ca^2+^ release of HAp, interfere with normal metabolic processes, and lead to tumor cell necrosis and apoptosis [25]. Bioceramics are brittle, resulting in poor mechanical strength. These bioactive ceramics are difficult to form; for example, the HAp degradation time is long, they are brittle, they have low mechanical strength, and HAp applications in load-bearing applications are limited.

A large number of studies in the literature have reported that graphene and its derivatives have the ability to assist bone regeneration [26], which is a promising bone tissue engineering material. Zhou K. [27] prepared 3D porous HA/rGO scaffolds with a hierarchical structure, suitable porosity and pore size, and good biomechanical strength. Supported rGO can improve cell adhesion, promote proliferation and spontaneous osteogenic differentiation of bone marrow mesenchymal stem cells (BMSCs), and greatly accelerate bone inward growth in scaffolds and bone repair of critical bone defects. Liu et al. [28] added 1.0% reduced graphene (rGO) to the hydroxyapatite coating on the surface of an implant and the proliferation and differentiation of osteoblasts on the surface of the mixed material were stronger than those on the surface of pure hydroxyapatite. In terms of ALP activity, the ALP activity of pure HA and HA-rGO composites was significantly higher than that of the blank group. The expression of ALP in the HA-1.0 wt% rGO composite was about twice that of pure HA. It can be concluded that the combined action of HAp and GO alters the physicochemical characteristics supporting cellular compatibility. GO and rGO may be cytotoxic to some extent, and their toxicity has been shown to be related to exposure time, dose and/or concentration, and surface chemistry [27].

ZnO nanostructures (ZnO NStrs) have strong biological activity. Studies have shown that using a small number of ZnONPs to promote cell growth, proliferation, and differentiation can promote tissue regeneration, angiogenesis, and bone integration processes [29,30]. It has been reported that ZnO composites exhibit excellent cell viability and cell proliferation, but when the zinc oxide content exceeds 10%, the results show that zinc oxide has long-term toxic effects [31]. Bhowmick et al. [32] prepared a chitosan-pegyl-nano-hydroxyapatite-ZnO composite scaffold, which supported the growth and proliferation of osteoblastic MG-63 cells. The study indicated that the surface area of the nanocomposite increased due to the addition of ZnONPs and nHAp. Protein adsorption on the mixed nanocomposite scaffold was higher than that of the control group, indicating better cell–matrix interaction, adhesion, and diffusion on the scaffold. An MTT test also showed that the viability of scaffold cells prepared by composite materials was higher due to the presence of nHAp. Zn^2+^ has been reported to have broad-spectrum antimicrobial and antifungal properties [33] due to its ability to enter microorganisms and become cytotoxic to prokaryotes. However, studies have shown that it exhibits a double reaction on endothelial cells, showing pro-angiogenic activity at a low concentration and opposite activity at a high concentration [34]. A number of studies have shown that the toxicity of ZnO nanoparticles is related to their dose and action time [8]. However, high concentrations of zinc ions can lead to toxicity, because excessive zinc can affect cellular zinc homeostasis and cause protein dysfunction [35]. In this study, the relative cell proliferation rate of CCK-8 in the 3% experimental group on the seventh day was lower than that in the other groups, showing a statistical difference from the control group.

### 3.4. Results of Antibacterial Experiment

**Figure 18:**
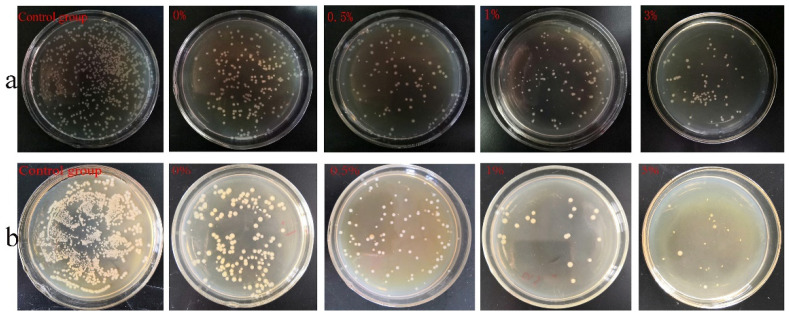
Colony count results: (**a**) *E. coli*, (**b**) *Staphylococcus aureus.*

**Figure 19:**
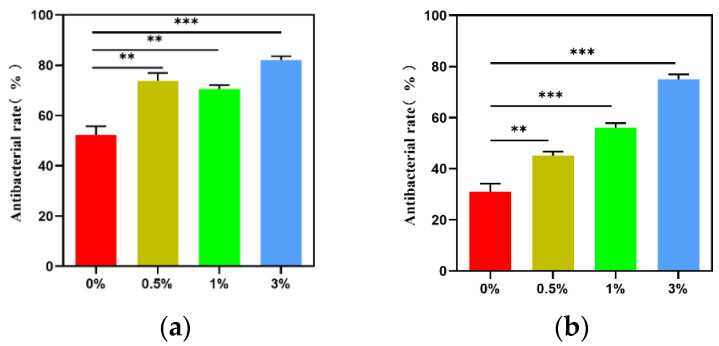
Antibacterial rate of composite microspheres in each group: (**a**) *Staphylococcus aureus*; (**b**) *E. coli*; ** *p* < 0.01; *** *p* < 0.001.

Figure 18 shows the formation of colonies in each group when cultured with *Escherichia coli* and *Staphylococcus aureus*, respectively. The composite microspheres of the 0.5%, 1%, and 3% groups had a good antibacterial effect when mixed with *Escherichia coli* and *Staphylococcus aureus*. Among the four experimental groups, the composite microspheres in the 3% group had the highest bacterial inhibition rate (Figure 19).

**Figure 20:**
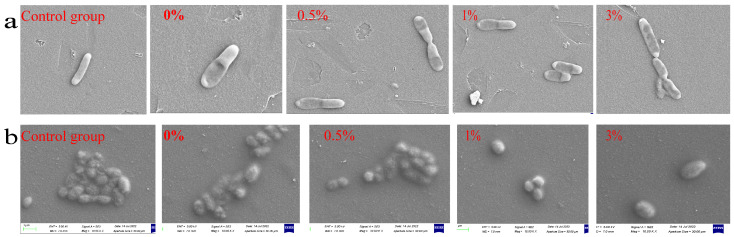
Morphology of microspheres co-cultured with bacteria: (**a**) *E. coli*, (**b**) *Staphylococcus aureus.*

The morphological changes in bacterial cell surface were observed using a scanning electron microscope (SEM) when composite microspheres were co-cultured with bacteria. By observing the scanning electron microscope image (Figure 20a), the normal *E.coli* in the control group was short and rod-shaped, with blunt round ends and a full shape. However, in the experimental group, *E. coli* showed cell shrinkage and a slightly rough surface, and this change was more obvious with the increase in GO-ZnO composite content. Figure 20b shows the bacterial changes after co-culture of composite materials and *Staphylococcus aureus*. There is no obvious difference in morphology between the control group and the experimental group, and *Staphylococcus aureus* is smooth and spherical. The difference is that in the same visual field, the number of *Staphylococcus aureus* in the experimental group is less than that in the control group, and the number of *Staphylococcus aureus* in the 0%, 0.5%, 1%, and 3% groups gradually decreases with the increase in the content of the GO-ZnO composite, indicating that the antibacterial effect is gradually enhanced. All of the above results show that GO/ZnO-HAp composite microspheres have a certain inhibitory effect on *Escherichia coli* and *Staphylococcus aureus*.

**Figure 21:**
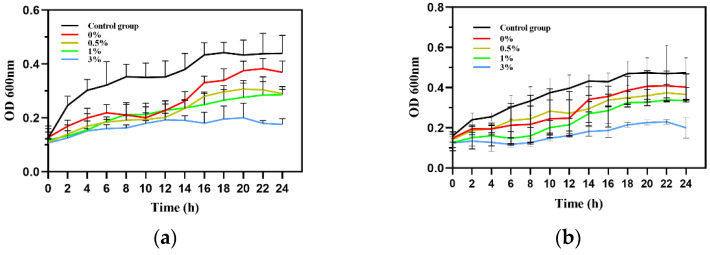
Bacterial growth curves: (**a**) *E. coli*, (**b**) *Staphylococcus aureus.*

As shown in Figure 21, during the 24 h measurement, the bacterial absorbance value of the control group increased significantly with time. In the experimental group, the growth of *Escherichia coli* and *Staphylococcus aureus* was inhibited to varying degrees under the action of GO/ZnO-HAp and the growth curve of the bacteria remained flat during the observation period of 24 h. As shown in Figure 21, compared with the blank control group, it can be observed that the experimental group significantly prolonged the time required for bacteria to enter the logarithmic growth phase and significantly inhibited the total bacterial growth, indicating that composite microspheres have a significant inhibitory effect on the growth of *Escherichia coli* and *Staphylococcus aureus*. With an increase in the proportion of GO-ZnO nanomaterials added, the antibacterial effect on *Escherichia coli* and *Staphylococcus aureus* gradually increased with the increase in their concentration, suggesting that they have a long-term antibacterial effect.

nHAp has antibacterial effects on both Gram-positive and Gram-negative bacteria. HA causes mechanical damage to the bacterial membrane due to its abrasive surface [36]. Electrostatic interactions between nHAp and bacterial cell walls can cause nanoparticles to enter the cytoplasm. HAp entering and recombining inside bacteria will lead to the death of the bacteria [37]. In addition, HAp increases oxidative stress by affecting the number of ROS inside and outside the cell. Oxidative stress attacks many of the cellular components needed for life within bacteria [38]. The antibacterial effect of nHAp was also reflected in this study. The colony count (Figure 18) and bacterial growth curve (Figure 21) showed that the antibacterial effect of nHAp was higher than that of the control group.

Raw graphene, GO, and reduced GO have been demonstrated to have antibacterial activity [39,40,41]. Studies have shown that among the carbon-based materials discussed so far, graphene oxide exhibits the most effective antibacterial activity at similar concentrations and culture conditions, followed by reduced graphene oxide, graphite, and graphite oxide [42]. Many mechanisms have been discovered and used to explain the antibacterial effects of graphene-based materials (GBMs). The most common antimicrobial mechanism is physical injury, where a vertical surface “blade” on the edge of the graphene sheet can insert and cut the membrane, resulting in cell dysfunction and leakage of cytoplasmic components, leading to antibacterial and bactericidal effects. Secondly, GBMs produce antibacterial effects by generating reactive oxygen species (ROS) and charge transfer. There are also antimicrobial mechanisms such as photocatalysis and destructive extraction of a large number of phospholipid molecules [43]. Contact with bacteria produces oxygen free radicals that denature bacterial proteins and deconstruct biofilms. This oxidative and stress-mediated antibiofilm activity suggests that rGO may prevent the reactivation of resistant bacteria in the treatment of trauma infections [44].

ZnO nanostructures have strong biological activity, and a large number of studies have shown that the use of a small amount of ZnONPs can promote cell growth, proliferation, and differentiation, as well as tissue regeneration, and promote angiogenesis and bone integration processes [29,30]. Zn^2+^ has been reported to have broad-spectrum antimicrobial and antifungal properties [33] because of its ability to enter microorganisms and become cytotoxic to prokaryotes. However, studies have shown that it exhibits a double reaction to endothelial cells, showing pro-angiogenic activity in low concentrations and the opposite in high concentrations. A number of studies have shown that the toxicity of ZnO nanoparticles is related to their dose and action time [8].

## 4. Conclusions

In summary, GO-ZnO-nHAp composite microspheres were prepared using the ionic gel-dropping method and protective atmosphere sintering. The microspheres have stable chemical properties, good hydrophilicity and biocompatibility, and certain antibacterial properties. However, bone regeneration materials need to be in long-term contact with tissues or body fluids after implantation to ensure that bone regeneration materials and tissues show good or harmonious biological behavior, so bone regeneration materials need to be improved in terms of biological evaluation before use. Part of the preparation process of the experimental material is relatively simple, and the preparation process can be improved later to prepare more uniform and unified microspheres in shape and size. This experiment only studied some cell biocompatibility items in vitro and more perfect experiments should be conducted in the future, such as cell adhesion, PCR determination of osteogenic-differentiation-related genes, in vivo animal experiments, and other evaluation methods, so as to provide more accurate data for the biological performance evaluation of composite microspheres.

## Figures and Tables

**Table 1:** Correspondence between cell proliferation rate and cytotoxic reaction grade.

Level	Relative Growth Rate
0	≥100
1	80–99
2	50–79
3	30–49
4	0–29

## Data Availability

Data is contained within the article.
